# Development of the external and internal shame scale: Japanese version

**DOI:** 10.1186/s13104-021-05698-2

**Published:** 2021-08-03

**Authors:** Yoichi Hiramatsu, Kenichi Asano, Yasuhiro Kotera, Ayumu Endo, Eiji Shimizu, Marcela Matos

**Affiliations:** 1 Research Center for Child Mental Development, Chiba University Graduate School of Medicine, 1-8-1 Inohana, Chuo-ku, Chiba, 260-8670 Japan; 2 The Japanese Centre for Compassionate Mind Research and Training, Tokyo, Japan; 3 Komachi Clinical Psychology Office, 2-11-1, Minamisaiwai, Nishi-ku, Yokohama-MS Bldg., Yokohama, Kanagawa 220-0005 Japan; 4 Department of Psychological Counseling, Faculty of Psychology, Mejiro University, 4-31-1 Nakaochiai, Shinjuku-ku, Tokyo, 161-0032 Japan; 5 National Center for Cognitive Behavior Therapy and Research, National Center of Neurology and Psychiatry, Kodaira, Japan; 6 Human Sciences Research Centre University of Darby, Kedleston Road, Derby, DE22 1GB United Kingdom; 7 Department of Psychology, Faculty of Letters, Komazawa University, 1-23-1, Komazawa, Setagaya-ku, Tokyo, 154-8525 Japan; 8 Department of Cognitive Behavioral Physiology, Chiba University Graduate School of Medicine, 1-8-1 Inohana, Chuo-ku, Chiba, 260-8670 Japan; 9 University of Coimbra, Faculty of Psychology and Educational Sciences, Center for Research in Neuropsychology and Cognitive Behavioral Intervention (CINEICC), Rua do Colégio Novo 3000-115 Coimbra, Portugal

**Keywords:** External shame, Internal shame, Depression, Fear of compassion

## Abstract

**Objective:**

Shame contains external and internal aspects. However, a Japanese language scale for simultaneously assessing both aspects of shame has not been developed to date. This study aimed to standardize the Japanese version of the External and Internal Shame Scale (EISS-J). An online survey was conducted among university students (*N* = 203) at six universities in Japan (Study 1). A retest questionnaire was sent to the participants by email three weeks after the first survey (Study 2). Study 1 examined the internal consistency, factor structure, and criterion-related validity of the EISS-J, while Study 2 examined its test-retest reliability. Moreover, an additional study was conducted to examine the criterion-related validity of the scale.

**Results:**

Study 1 demonstrated the high internal consistency of the EISS-J. Moreover, confirmatory factor analysis indicated a two-factor model: external and internal shame. However, exploratory factor analysis indicated a three-factor structure. Study 2 confirmed the test-retest reliability of the scale. Furthermore, both studies indicated correlations between the EISS-J and fear of compassion, anger, humiliation, depression, anxiety, and stress. In addition, the study established the criterion-related validity of the scale. These results confirmed adequate reliability and validity of the EISS-J.

**Supplementary Information:**

The online version contains supplementary material available at 10.1186/s13104-021-05698-2.

## Introduction

Shame comprises two dimensions [1]. Internal shame is related to the internal dynamics of the self and how the self judges and feels about itself [2], including tendencies to focus on negative aspects of the self and maintain global self-judgments of being bad, inferior, and flawed [3–5]. This type of shame is related to negative self-evaluations and self-directed affects, such as feelings of self-disgust [2]. On the other hand, external shame is associated with the perception that others have bad impressions of oneself, such as being inferior or worthless [6]. When experiencing external shame, people engage in defensive maneuvers, with the behavior oriented toward trying to positively influence one’s image in the minds of others inferior and worthless [6].

External and internal shame are regarded as different dimensions of the identical emotional experience, which are closely linked and encompass the same core domains of inferiority/inadequacy, exclusion, emptiness, and criticism [7]. Both aspects of shame are crucial for social functioning [7].

Until recently, there have been no scales for simultaneously assessing these two dimensions of shame. Recently, the External and Internal Shame Scale (EISS) for assessing external and internal shame was developed [7]. The present study aimed to develop a Japanese version of the EISS and verify its validity and reliability.

## Main text

### Materials and methods

#### Participants

A questionnaire was administered to university students (N = 203) from six universities in the metropolitan area of Japan, and the responses of participants without missing data were analyzed (N = 202). Many of them were undergraduates, but some were graduate students. The participants included 54 men, 146 women, and two respondents who did not disclose their gender. Their age ranged from 18 to 28 years, with a mean age of 20.09 years (*SD* = 1.78).

### Measures

#### Japanese version of the External and Internal Shame Scale (EISS-J)

The EISS contains eight items rated on a 5-point Likert scale ranging from 0 to 4, with four items assessing external shame and four assessing internal shame. The original EISS contained the following core domains of shame experiences: (1) inferiority/inadequacy, (2) sense of isolation/exclusion, (3) uselessness/emptiness, and (4) criticism/judgment, resulting in a pool of 16 initial items. Subsequently, researchers selected one pair of items from the pool that adequately expressed each domain. Consequently, a scale consisting of final eight items was developed (example items in the inferiority/inadequacy domain included "I am different and inferior to others" and "People around me see me as not being up to their standards") [7]. The English version was translated into Japanese by two experts (YH and YK) after obtaining the original authors' approval. Back-translations were conducted by two native English speakers from a translation company (Crimson Interactive Japan Co., Ltd.). The two retranslated English versions were integrated, and the resulting version was sent to the original author, who confirmed the homogeneity of the Japanese version of the scale.

#### Japanese version of the Other as Shamer Scale (OAS)

The OAS was developed to assess external shame [8]. The Japanese version is composed of 18 items rated on a 5-point Likert scale ranging from 0 to 4, which are similar to the original version [9]. A strong positive correlation was reported between the EISS and the OAS [7].

#### Japanese version of the State-Trait Anger Expression Inventory (STAXI)

The STAXI was developed to measure anger [10]. The Japanese version, which contains ten items of the Trait Anger scale rated on a 4-point Likert scale ranging from 1 to 3, was used in the present study [11]. A positive correlation between anger and external shame was confirmed [12]. A total of 106 participants responded to the STAXI, as the STAXI was added midway through the study to reinforce the examination of the validity of external shame.

#### Japanese version of the Fear of Compassion Scale (FC)

The FC [13] and its Japanese version [14] are composed of three subscales that include 38 items: fear of compassion for others (FCforO, ten items), from others (FCfromO, 13 items), and for self (FCforS, 15 items). This scale is rated on a 5-point Likert scale ranging from 0 to 4. Fear of compassion is the sense of resistance to expressing compassion for others or receiving compassion from others. Previous research noted shame memories as a background to the fear of compassion, suggesting that shame experiences increase depression and anxiety, mediated by fear of compassion for and from others [15].

#### The Japanese version of the Beck Depression Inventory-Second Edition (BDI-II)

The BDI-II [16] and its Japanese version [17] assess depression. The high validity and reliability of the scale for assessing depression have been confirmed [17]. The scale contains 21 items rated on a 4-point Likert scale ranging from 0 to 3.

#### Japanese version of the Humiliation Inventory (HI-J)

Humiliation, like shame, is a form of self-consciousness emotion [18]. It has been shown that during the experience of shame, there is an aggressive aspect that is focused on others. [18]. A positive correlation was predicted between the HI-J and the EISS-J by using the 12 items of cumulative humiliation rated on a 5-point Likert scale ranging from 1 to 5.

#### Japanese version of the Depression, Anxiety, Stress Scale-15 (DASS-15)

The DASS-15 consists of 15 items rated on a 4-point Likert scale ranging from 0 to 3 [19] and is used to assess depression, anxiety, and stress. A positive correlation was demonstrated between the DASS-21 and the EISS [7]. Therefore, the present study predicted a positive correlation between the DASS-15 and the EISS-J.

### Procedures

An online questionnaire was conducted with university students enrolled in six Japanese universities that agreed to participate in the research. Participants were recruited by providing a link to the survey to students attending online lectures and one university also on the universities’ subject recruitment websites. The survey was conducted between July and November 2020. Responses were anonymous. Participants were provided with written explanations regarding the survey's purpose, personal information protection, confidentiality, and arbitrariness of responses. Afterwards, participants were instructed, "If you agree to complete the survey after reviewing the above instructions, please click the 'Answer' button below to proceed and answer the questions. This study was approved by the ethics committee of Chiba University (No. 3441). The number of accesses to the site was 577, and the number of respondents was 203 (35.18%). However, test accesses and multiple accesses by the same person are included. Emails were sent to the participants three weeks after the survey, requesting them to participate in the online retest study to identify the scale's test-retest reliability. The emails contained the URL of the retest questionnaire. Participants were able to respond on the same day or later. All respondents completed the survey within five weeks of the initial survey, except for one who completed it eight weeks after the initial survey. The method for obtaining informed consent was the same as that in Study 1. Statistical analyses were conducted using SPSS and AMOS software.

## Results

The factor structure of the eight-item EISS-J was examined using confirmatory factor analysis (CFA) by assuming a higher-order factor (global shame) and two lower-order factors (external shame and internal shame), similar to the original version. However, a satisfactory level of fit rate could not be obtained (Additional file 2: Figure S1); therefore, exploratory factor analysis (EFA) was implemented. Three factors were identified based on eigenvalue decay (4.001, 0.941, 0.911, 0.626), unlike the original factor structure. The first and second factors were extracted as external and internal shame, respectively; however, out of the four shame domains, only the sense of isolation/exclusion was extracted separately, forming the third factor. This factor was called Isolation (Additional file 1: Table S1). The CFA with error covariance between Items 2 and 3 relating to the isolation factor was conducted again using EFA results as a reference (Additional file 2: Figure S1), which improved the fit rate (Table 1). Subsequent analyses were conducted using a higher-order factor and two lower-order factors, external shame (EISS-J-ES) and internal shame (EISS-J-IS), based on the CFA results, which was consistent with the original and allowed for cross-national comparisons.

**Table 1:** Goodness-of-fit statistics of EISS-J

	χ2	df	CMIN	GFI	AGFI	CFI	TLI	RMSEA
Higher-order factor model Higher-order (with error covariance)	103.398 58.252	19 18	p < 0.001 p < 0.001	0.888 0.935	0.788 0.869	0.864 0.935	0.791 0.899	0.148 0.105
Original model (Ferreira et al. [7])	126.73	19	p < 0.001	0.96	–	0.96	0.94	0.09

The reliability of the EISS-J was examined using internal consistency and test-retest methods. Cronbach's alphas for all scales were shown in Additional file 3: Table S2.

A correlation analysis was conducted between the EISS-J and its subfactors (EISS-J-ES and EISS-J-IS) and related scales to examine the criterion-related validity of the EISS-J. The results are presented in Table 2. A strong positive correlation was found between the EISS-J and the OAS, the FC, and the BDI-II. The two subfactors of the EISS-J demonstrated a significant positive correlation with the OAS, which was consistent with the original [7]. As in previous studies [15], the FC showed a high positive correlation with centrality of shame memory. However, only one strong correlation was not found between fear of compassion for others and internal shame. In addition, a significant positive correlation was observed between the STAXI and the EISS-J. However, there was no strong correlation between the STAXI and the EISS-J-ES, as predicted.

**Table 2:** Correlations between the EISS-J and oher scales

	EISS-J	ES	IS	OAS	FC	FCforO	FCfromO	FCforS	STAXI
ES	0.658**								
IS	0.625**	0.713**							
OAS	0.832**	0.588**	0.482**						
FC	0.686**	0.451**	0.412**	0.630**					
FCforO	0.243**	0.187**	0.086	0.287**	0.661**				
FCfromO	0.691**	0.499**	0.438**	0.637**	0.885**	0.460**			
FCforS	0.637**	0.365**	0.417**	0.542**	0.896**	0.378**	0.686**		
STAXI	0.287**	0.017	−0.048	0.341**	0.351**	0.369**	0.221*	0.307**	
BDI-2	0.651**	0.396**	0.469**	0.608**	0.637**	0.241**	0.583**	0.655**	0.352**

EISS-J, External and Internal Shame Scale Japanese version: ES, external shame: IS, internal shame: Isol, Isolation: OAS, Other As Shamer scale japanese version: FC, Fear of Compassion scale: FCforO, fear of compassion for other: FCfromO, fear of compassion from other: FCforS, fear of compassion for self: STXI, State-Trait Anger Expression Inventory (trate anger items)

**p < 0.01. *p < 0.05

### Study 2

A retest study was conducted using an online questionnaire three weeks after Study 1 to examine the scale's test-retest reliability and criterion-related validity.

A total of 84 students (20 men, 62 women, and two respondents who did not disclose their gender) participated in this study. Their age ranged from 18 to 28 years (mean age = 20.37, *SD* = 1.94).

The results indicated a high test-retest correlation (EISS-J: r = 0.83, EISS-J-ES: r = 0.77, EISS-J-IS: r = 0.82) and test-retest reliability of the scale. Cronbach's alphas for all scales were shown in Additional file 4: Table S3. Moreover, correlation analysis conducted on the HI-J and the DASS-15 indicated a high positive correlation between the DASS-15 and the EISS-J, suggesting a correlation between shame and mental health symptoms, such as depression, anxiety, and stress. However, there was no significant correlation between the HI-J and the EISS-J (Table 3).

**Table 3:** Correlations between the EISS-J, DASS and HI-J

	EISS-J	ES	IS	DASS
ES	0.907**			
IS	0.929**	0.687**		
DASS	0.514**	0.462**	0.474**	
HI-J	0.182	0.210	0.129	0.499**

EISS-J, External and Internal Shame Scale Japanese version: ES, external shame: IS, internal shame: DASS, Japanese version of the Depression, Anxiety, Stress Scale-15: HI-J, Japanese version of the Humiliation Inventory (Cumulative Humiliation)

**p < 0.01. *p < 0.05

## Discussion

The CFA results identified two EISS-J factors. The EISS-J included both internal and external shame factors, similar to the original version. However, the EFA extracted isolation as a separate factor. Shame includes negative self-cognition [2, 8]; however, the sense of isolation may have encompassed pure isolation that did not include critical self-recognition. For instance, meeting other people was restricted at the time of the study due to the COVID-19 pandemic. Therefore, people may have faced increased loneliness not associated with criticism or exclusion from others.

Examining correlations between the other EISS-J scales indicated that the EISS-J, similar to the original scale, was strongly correlated with various symptoms of mental health, such as depression, stress, anxiety, and fear of compassion. Moreover, the results of criterion-validity studies were partly different, although they were generally consistent with the predictions. No significant correlation was found between external shame and the STAXI. Similarly, external shame was not significantly correlated with humiliation, which was related to aggressive emotions focused on others, such as resentment and the desire to take revenge. Anger and humiliation are aggressive feelings toward others. Shame has a submissive aspect (damage limitation), which is a strategy to reduce attacks and exclusion by others [2]. In contrast, anger is an emotion expressed by attacking others or destroying targets [11]. External shame factors of the EISS-J may more closely reflect the aspects of submissive strategies. The relationship between the EISS-J and feelings of aggression toward others, such as anger and humiliation, requires further investigation.

The findings confirmed the adequate reliability and validity of the EISS-J for simultaneously assessing external and internal shame. This study validated the three-factor model using the EFA and a higher-order factor model using the CFA. The higher-order factor model could be used for future international studies, while the three-factor model could be used for studies in Japan.

## Limitations

The participants in this survey were healthy university students. In addition, as many participants were women, the gender distribution was disproportionate. Therefore, a gender bias may have occurred. Moreover, the retest study contained a relatively small sample size. Future studies should investigate extensive and diverse participants to confirm the results of this study. Furthermore, the model should be re-examined with the CFA based on larger datasets.

## Supplementary Information

**Additional file 1: Table S1.** Factor loading for the EISS-J

**Additional file 2: Figure S1.** Results of confirmatory factor analysis of EISS-J

**Additional file 3: Table S2.** The cronbach’s α for all scale in study 1.

**Additional file 4: Table S3.** The cronbach’s α for all scale in study 2.

## Data Availability

The datasets generated and analyzed during the current study are available from the corresponding author on reasonable request.

## Abbreviations

CFA: Confirmation factor analysis
EFA: Exploratory factor analysis
EISS: External and Internal Shame Scale
EISS-J: External and Internal Shame Scale—Japanese version
OAS: Other as Shamer Scale—Japanese version
STAXI: State-Trait Anger Expression Inventory—Japanese version
FC: Fear of Compassion Scale—Japanese version
BDI-II: Beck Depression Inventory, Second Edition—Japanese version
HI-J: Humiliation Inventory—Japanese version
DASS-15: Depression, Anxiety, Stress Scale-15—Japanese version
