# Supplementary figures and images for: Development of the external and internal shame scale: Japanese version
Source: BMC Res Notes. 2021 Aug 3;14:297. doi: 10.1186/s13104-021-05698-2 (PMC8329630; doi:10.1186/s13104-021-05698-2)

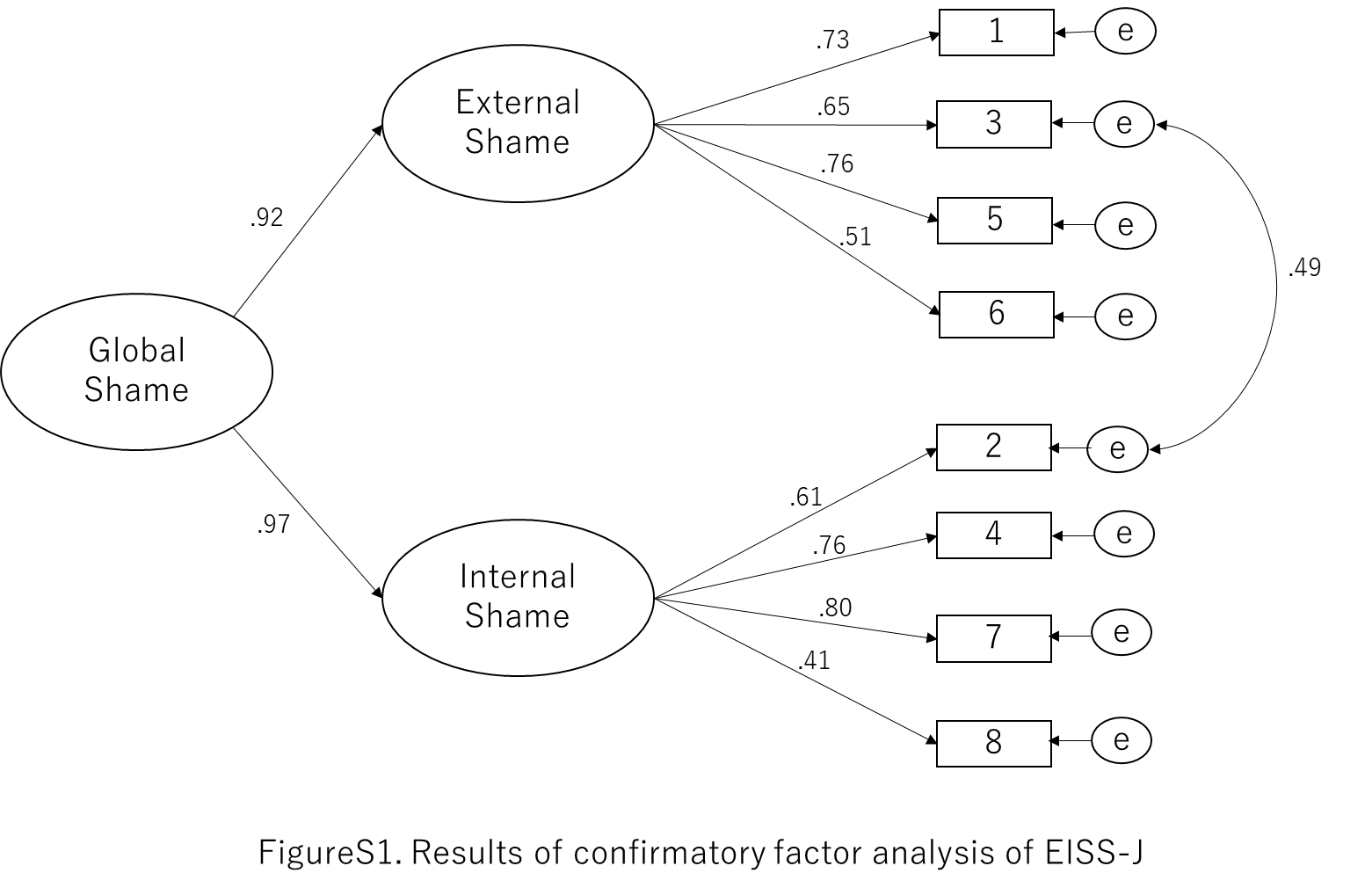

Supplement: Supplementary file 2 — Additional file 2: Figure S1. Results of confirmatory factor analysis of EISS-J [file 13104_2021_5698_MOESM2_ESM.docx]
