# Supplementary material for: Development of the external and internal shame scale: Japanese version
Source: BMC Res Notes. 2021 Aug 3;14:297. doi: 10.1186/s13104-021-05698-2 (PMC8329630; doi:10.1186/s13104-021-05698-2)
Supplement: Supplementary file 2 [file 13104_2021_5698_MOESM1_ESM.docx]

TableS1. Factor loadings for the EISS-J

	Factor1 ES	Factor2 IS	Factor3 Isol
EISS6	.824		
EISS1	.669		
EISS5	.569		
EISS4		.779	
EISS7		.696	
EISS8		.439	
EISS3			.891
EISS2			.526
