# Supplementary material for: Development of the external and internal shame scale: Japanese version
Source: BMC Res Notes. 2021 Aug 3;14:297. doi: 10.1186/s13104-021-05698-2 (PMC8329630; doi:10.1186/s13104-021-05698-2)
Supplement: Supplementary file 3 [file 13104_2021_5698_MOESM3_ESM.docx]

TableS2. the Cronbach's α for all scales in study1

	EISS-J	ES	IS	OAS	FC	FCforO	FCfromO	FCforS	STAXI	BDI-2
Cronbach's α	.85	.77	.71	.92	.94	.82	.90	.93	.83	.92

EISS-J, External and Internal Shame Scale japanese version: ES, external shame: IS, internal shame: Isol, Isolation: OAS, Other As Shamer scale japanese version: FC, Fear of Compassion scale: FCforO, fear of compassion for other: FCfromO, fear of compassion from other: FCforS, fear of compassion for self: STXI, State-Trait Anger Expression Inventory(trate anger items)
