# Supplementary material for: Development of the external and internal shame scale: Japanese version
Source: BMC Res Notes. 2021 Aug 3;14:297. doi: 10.1186/s13104-021-05698-2 (PMC8329630; doi:10.1186/s13104-021-05698-2)
Supplement: Supplementary file 4 [file 13104_2021_5698_MOESM4_ESM.docx]

TableS3. The Cronbach's α for all scales in study2

	EISS-J	ES	IS	DASS	HI-J
Cronbach's α	.87	.79	.79	.90	.94

EISS-J, External and Internal Shame Scale japanese version: ES, external shame: IS, internal shame: DASS, Japanese version of the Depression, Anxiety, Stress Scale-15: HI-J, Japanese version of the Humiliation Inventory(Cumulative Humiliation)
